# Researches on cognitive sequelae of burn injury: Current status and advances

**DOI:** 10.3389/fnins.2022.1026152

**Published:** 2022-11-04

**Authors:** Chenchen Xie, Jun Hu, Yong Cheng, Zhongxiang Yao

**Affiliations:** ^1^Department of Neurology, Affiliated Hospital and Clinical Medical College of Chengdu University, Chengdu, China; ^2^Department of Neurology, The Second Affiliated Hospital of Chongqing Medical University, Chongqing, China; ^3^Department of Neurology, First Affiliated Hospital of Army Medical University, Chongqing, China; ^4^Department of Neurology, General Hospital of Central Theater Command of PLA, Wuhan, China; ^5^Department of Physiology, Army Medical University, Chongqing, China

**Keywords:** burn injury, cognitive dysfunction, neuroinflammation, blood-brain barrier (BBB), hormone imbalance, memory defect

## Abstract

Burn injury is a devastating disease with high incidence of disability and mortality. The cognitive dysfunctions, such as memory defect, are the main neurological sequelae influencing the life quality of burn-injured patients. The post-burn cognitive dysfunctions are related to the primary peripheral factors and the secondary cerebral inflammation, resulting in the destruction of blood-brain barrier (BBB), as is shown on Computed Tomography (CT) and magnetic resonance imaging examinations. As part of the neurovascular unit, BBB is vital to the nutrition and homeostasis of the central nervous system (CNS) and undergoes myriad alterations after burn injury, causing post-burn cognitive defects. The diagnosis and treatment of cognitive dysfunctions as burn injury sequelae are of great importance. In this review, we address the major manifestations and interventions of post-burn cognitive defects, as well as the mechanisms involved in memory defect, including neuroinflammation, destruction of BBB, and hormone imbalance.

## Introduction

Burn injury is the fourthly prevalent injury in the world with poor survival (Mashreky et al., 2018), and is the third leading cause of preventable death in children (Chong et al., 2020). The top three risk factors for the poor survival of burn injury are the advanced age, multiple coexisting comorbidities, and the increased percentage of burn area to total body surface area (%TBSA) (Brusselaers et al., 2010).

Burn injury induces both the local and the systemic damages. Treatment of complications caused by burn injury is related to its prognosis, and is even a life or death matter (Smolle et al., 2017). The main complications include shock, wound infection, pulmonary infection and injury, acute renal insufficiency, neurological sequelae, multiple organ failures. Brain is one of the remote organs affected by burn injury (Halm et al., 2006; Nielson et al., 2017). Neurological complications such as persistent headache, memory loss, and paresthesia were reported in the survivors of the 2013 nightclub fire accident (Martins de Albuquerque et al., 2013). The morbidity of central nervous system (CNS) was significantly increased after burn injury (Vetrichevvel et al., 2016). One study showed that hospitalized burn injury patients had over twice as many admissions for a nervous system condition as non-burn injury control cohort, and their length of stay was 3.25 times longer than that of control cohort (Vetrichevvel et al., 2016). Therefore, treatment for the neurological sequelae is closely related to the prognosis of burn-injured patients.

**FIGURE 1:**
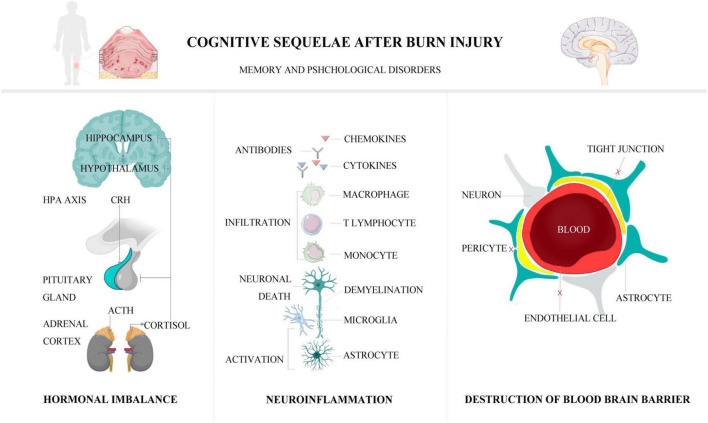
The pathophysiological mechanisms of post-burn cognitive sequelae.

The main neurological sequelae following burn injury are cognitive dysfunctions, including memory defects, amnesia, dementia, depression, anxiety, post-traumatic stress disorder (PTSD), hallucinations, and delirium (Wollgarten-Hadamek et al., 2009). Although other neurological sequelae including peripheral neuropathies, chronic neuropathic pain, post-burn pruritus, and acute/chronic fatigue are also noted (Chung et al., 2020; Boersma-van Dam et al., 2021a; Shu et al., 2022), memory defects and psychological impairments as the most common clinical manifestations of cognitive dysfunctions following burn injury are the main points to be covered in this article (Purohit et al., 2014). This review elaborates the mechanisms of memory defects following burn injury, such as neuroinflammation, destruction of blood-brain barrier (BBB), and hormone imbalance. The main clinical presentations, management, and outcomes of post-burn cognitive impairments are also addressed. A visible graphical abstract that demonstrates the cognitive sequelae of burn injury and mechanisms is provided (Figure 1).

## The pathophysiologic mechanisms of post-burn cognitive sequelae

**FIGURE 2:**
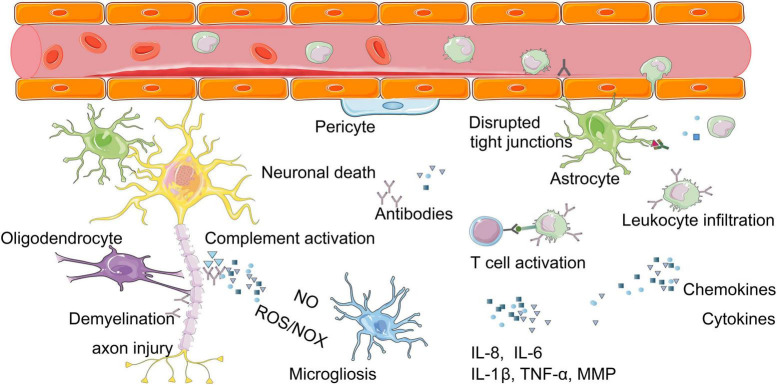
Molecular mechanisms of neuroinflammation after burn injury. Burn injury can cause a series of inflammatory reactions in brain, for example, the release of inflammatory mediators (chemokines, cytokines), the imbalance of immune system (T cell activation, complement activation). These above can lead to changes such as neuronal cell death, demyelination, axon injury, glial cell activation. ROS, reactive oxygen species; NOX, nitric oxide; IL-8, interleukin-8; IL-6, interleukin-6; IL-1β, interleukin-1 beta; MMPs, matrix metalloproteinases.

**FIGURE 3:**
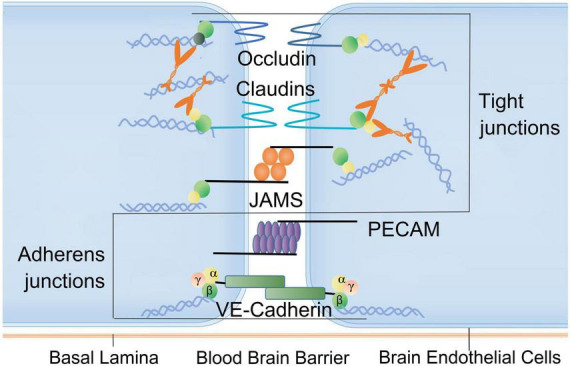
Factors associated with blood-brain barrier disruption after burn injury. Brain endothelial connections (BECs): the BBB is tightly connected by capillary endothelial cells. Tight junctions are composed of occludin family proteins, claudin and junction adhesion molecules (JAMs). The connections between endothelial cells also include adhesion junctions consisting of platelet endothelial cell adhesion molecules (PECAMs) and VE-cadherin. These structures and molecules can be damaged following burn injury. JAMs, junction adhesion molecules; PECAMs, platelet endothelial cell adhesion molecules.

**FIGURE 4:**
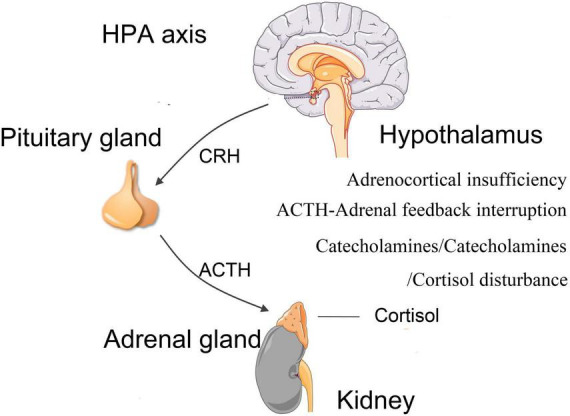
Hormonal imbalance. Burn injury can lead to dysregulations of hypothalamus-pituitary-adrenal (HPA) axis and abnormal secretions of cortisol and other hormones, such as adrenocortical insufficiency, upregulation of catecholamine and corticotrophin, downregulation of cortisol. HPA, hypothalamus-pituitary-adrenal; CRH, corticotropin-releasing hormone; ACTH, adrenocorticotropic hormone.

Factors affecting cognitive dysfunction after burn injury include primary and secondary ones. The primary factors are involved in the burn event itself and its treatment process. The secondary factors mainly refer to the indirectly secondary brain damage leading to memory impairments, such as inflammatory reactions in brain, destruction of BBB, and hormone imbalance (Figures 2–4; Flierl et al., 2009).

### Primary factors for post-burn cognitive impairments

In cases of severe burn injury, homeostasis and the interaction between the wound and the host regarding circulation and metabolism need to be controlled as life-threatening conditions (Paggiaro et al., 2022). Three primary factors are related to cognitive dysfunction following burn injury (Hendricks et al., 2017). The first one is process of burn event, including hypoxia and inhalation of toxic fumes (Lawrence et al., 2012). The second one is the multi-organ and multi-system responses caused by burn injury, including fluid and electrolyte imbalance, renal insufficiency, infection of local tissues (Mater et al., 2020). The third one is adverse treatment process, including abnormal absorption of topical drugs, pain medications, inappropriate fluid replenishment, malnutrition, and adverse pharmacological reactions due to the use of benzodiazepines, anesthetics, or painkillers (Purohit et al., 2014).

### Secondary factors for post-burn cognitive impairments

The secondary factors mainly include neuroinflammation, destruction of BBB, and hormone imbalance (Figure 2; Flierl et al., 2009). The acute systemic inflammatory response syndrome following burn injury is characterized by elevated levels of circulating pro-inflammatory cytokines and the activation of complement. Such cytokine storm, together with the systemic immune reactivity generated by the elevation of myriad of self-contained autoantigens, penetrate the BBB. All these “new invaders” invade the brain parenchyma and induce the glial cell activation (Berlanga-Acosta et al., 2020). This invasion causes neuroinflammation, along with the nitric oxide imbalance, oxidative stress and hormonal imbalance in the brain, leading to neuronal degeneration, apoptosis, abnormal neurotransmitters and transmission, dysfunction of neurovascular units (NVU), synaptic disruption, and consequently the defects of cognitive functions (Halm et al., 2006).

#### Neuroinflammation

Neuroinflammation is involved in the entire progress of cognitive dysfunctions after burn injury (Figure 2). An inflammatory response developed in the CNS after burn injury with at least 20% TBSA (Agay et al., 2008), as manifested by the sharp increase of pro-inflammatory cytokines at 3 h after burn injury (Reyes et al., 2006; Agay et al., 2008; Flierl et al., 2009). Abnormally high levels of cytokines in the brain are associated with morbidity and mortality of post-burn patients (Flierl et al., 2009). The serum levels of tumor necrosis factor-α (TNF-α), interleukin-1 beta (IL-1β), and interleukin-6 (IL-6) were reported to increase after burn injury in both human beings and animals (Gatson et al., 2009). In particular, the up-regulated level of TNF-α in brain is considered to be a common detrimental factor mainly damaging microvasculature where the cytokines and adhesion molecules are expressed (Mark and Miller, 1999). The level of prostaglandin E2 in cerebrospinal fluid is also increased after burn injury (Ozaki-Okayama et al., 2004). Neuroinflammation is accounted for by an imbalanced immune system after burn injury due to the following three changes.

Firstly, burn injury promotes the infiltration of immunocytes through BBB into brain tissues, among which the monocytes, circulating antigen-presenting leukocytes, play an important role in inflammation, phagocytosis, T cell differentiation, and innate immunity (Nahrendorf et al., 2010). Many activated monocytes were reported to infiltrate into brain tissues in mice (Zhang et al., 2013). Meanwhile, a large number of phagocytes and macrophages have also been found to infiltrate into brain tissues after burn injury (Gatson et al., 2009; Reyes et al., 2009). The activated phagocytes release a large number of proteases, reactive oxygen species (ROS), nitrogen oxide (NOX), and other pro-inflammatory mediators, which aggravate the neuroinflammatory responses after burn injury (Charo and Ransohoff, 2006). The macrophages increased oxidative metabolism after burn injury, as was shown by an increased activity of oxygen free radicals in burn victims (Schwacha and Somers, 1999).

Secondly, burn injury activates the complement system. Moderate complement activation is beneficial, but over-activation is harmful (Stahel and Barnum, 2006). The expression of C5a receptor (C5L2) was regulated following a standardized 30% TBSA full-thickness burn injury (Rittirsch et al., 2008) and was significantly upregulated at 24 h after burn injury, indicating that C5L2 is a functional receptor involved in the complement-mediated post-burn neuroinflammation (Flierl et al., 2009).

Thirdly, burn injury generates severe suppression of immune system. The dynamic changes of splenic T cells were detected after burn injury and were involved in the immunosuppression, indicating T cell homeostasis disorders (Patenaude et al., 2005). Immunocyte apoptosis is the main cause of immune suppression in the pathogenesis of burn injury (Huang et al., 2019). Evidence shows that the TNF-α-induced protein 8-like2 (TIPE2) is an immunosuppressive protein that participates in the apoptosis and pathogenesis of CD4^+^ T cells after burn injury, and is closely related to abnormal immune functions (Cao et al., 2021).

The CNS inflammatory response after burn injury is mediated by the peripheral release of damage associated molecular patterns (DAMPs), similar to that following surgical injury. However, the inflammatory response triggered by burns can last up to 3 years (Jeschke et al., 2011), which may lead to depletion of cytoprotective mechanisms, such as those mediated by heat shock protein72 (HSP72), and subsequently exacerbation of cognitive dysfunction (Nikolakopoulou et al., 2019). Research found burn injury can induce suppression of the oxidative to reductive nicotinamide adenine dinucleotide (NAD + /NADH) ratio, as well as oxidative stress and depletion of adenosine triphosphate (ATP) in skeletal muscle (Nakazawa et al., 2022). In the central nervous system, ATP depletion in brain tissue can occur in disease states, which can trigger inflammation and oxidative stress as well as mitochondrial dysfunction in the brain. The hippocampus, an important tissue for learning and memory, is susceptible to damage from inflammation and oxidative stress, which can affect synaptic plasticity and cognitive function (Spagnuolo et al., 2020). In addition, ATP depletion and associated inflammatory responses may lead to deposition of Aβ proteins and possibly also be associated with toxic Aβ conformation and p-tau level (Illes, 2020; Spagnuolo et al., 2020). However, there is a lack of studies directly related to ATP depletion in brain tissue after burn injury. Therefore, it is of great significance to investigate whether burn injury leads to inflammatory response, oxidative stress and ATP depletion in the brain, and the interactions between these alterations on the cognitive function after burn injury.

#### Destruction of blood-brain barrier

The BBB, composed of tight junctions between brain endothelial cells (BECs), basal lamina, perivascular pericytes and astrocytes, is the core part of NVU existing between circulatory system and brain parenchyma (Kaplan et al., 2020). The BBB controls the permeation of many substances between blood and brain parenchyma, the exchanges between CNS and its surrounding tissues, as well as the regulations of CNS homeostasis (Figure 3). Nevertheless, this barrier may be severely damaged after burn injury (Shabir et al., 2018; Table 1).

**TABLE 1:** Researches related to the blood-brain barrier dysfunction after burn injury.

Author	Research subjects	Burn model	Concerns	Results
Barone et al., 1997	Adult Sprague-Dawley rats	A third degree burn affecting 70% TBSA	The albumin leak in the cerebral vessels	Thermal injury led to an increased permeability of cerebral vessels.
Randolph et al., 2019	Female merino sheep	A third degree burn affecting 40% TBSA combined with smoke inhalation	BBB integrity and hemorrhage	Third-degree skin burn injury combined with smoke inhalation produced massive cerebral hemorrhaging, and BBB dysfunction characterized by congested and dilated blood vessels, as well as neutrophil infiltration into the brain.
Berger et al., 2007.	Adult Sprague-Dawley rats	A third degree burn affecting 70% TBSA	BBB integrity, brain edema, and MMP levels in the brain	The significant increase in cerebral permeability after serious systemic thermal injury could be related to early expression of MMPs.
Swann et al., 2007.	Adult Sprague-Dawley rats	A third degree burn affecting 60–70% TBSA	Brain edema and MMPs levels	The increase in MMP-9 expression and activity, associated with increased BBB permeability following thermal injury, indicates that MMP-9 may contribute to observed cerebral edema in peripheral thermal injury.
Li et al., 2009	Male mongrel dogs	A third degree burn affecting 50% TBSA	The manifestation of MRI and histopathology; changes of brain water content; the shapes and distribution of the cerebral capillaries; the volume, surface, and length fractions of cerebral capillaries	The changes of cerebral blood flow might play an important role in the pathogenesis of brain edema in the early stage of severe burn.
Patel et al., 2008	Adult male Sprague–Dawley rats	A third degree burn affecting 60–70% TBSA	BBB dysfunction; levels of tPA and uPA in the brain	Peripheral thermal injury induced an increase in the permeability of the BBB, which may be related with the increased expression of tPA and uPA,
Liu et al., 2009	Adult male Sprague–Dawley rats	A third degree burn affecting 30% TBSA	BBB permeability; brain edema; GFAP expression in the brain	The increase in GFAP expression may be associated with the increased BBB permeability following thermal injury.
Liu et al., 2011	Adult male Sprague–Dawley rats	A third degree burn affecting 30% TBSA	BBB permeability; the expression levels of ZO-1	The increased permeability of BBB after severe burns in the rat model may be related to the decreased expression of ZO-1.
Wang and Yang, 2005	Adult male Sprague–Dawley rats	A third degree burn affecting 40% TBSA	BBB permeability and brain edema	Burn injury induced an increase in the permeability of the BBB, leading to brain edema.
Moati et al., 1979	Burn injured patients; Rats	A third degree burn affecting 30% TBSA	BBB permeability	BBB permeabilizing activity was related to collagenolytic activity.
Yang et al., 2020	Wild-type C57BL/6J female mice	A third degree burn affecting 30% TBSA	Transcellular vesicular transport (transcytosis) in BBB; neuroinflammtion; tight junction proteins (TJs)	Burns could induce an increase in the permeability of BBB via paracellular pathway as well as transcytosis. Umbilical cord-derived mesenchymal stem cells could protect the integrity of BBB after burns by protecting the tight junction, as well as reducing transcytosis and neuroinflammation.
Reyes et al., 2009	Adult male Sprague–Dawley rats	A third degree burn affecting 70% TBSA	BBB permeability and neuroiflammation	The expression of TNF-α, IL-1β and ICAM-1 were elevated at 3 h and remained high at 7 h following burn injury.
Jiang et al., 2021	Adult male Sprague–Dawley rats	A third degree burn affecting 30% TBSA	Levels of HIF-1α, Claudin5, ZO-1, and VE-cadherin	Burn injury enhanced vascular permeability, resulting in the disruption of endothelial cell junction integrity.
Basch and Fazekas, 1970	Wistar rats	A third degree burn affecting 20% TBSA	BBB permeability	BBB permeability was considerably ruptured even 5 days after burn injury.
Khan et al., 2020	Male albino mice	A burn injury affecting 25% TBSA	Cognitive behaviors; the JNK/caspase-3 and BDNF/VEGF levels; pro-inflammatory cytokine; levels of antioxidant, nitric oxide, and corticosterone	The cognitive behavior defects after burn injury may be related with the modulation of JNK-mediated inflammation, oxidative stress, apoptosis, and BDNF/VEGF signaling in an acute mouse model with burn injury.
Cherry et al., 2013	C57BL/6J female mice	A full thickness burn affecting 10% TBSA combined whole-body gamma irradiation	Learning and memory capacity; vascular activation; neuroinflammation; neurogenesis	Thermal injury lowered the threshold for radiation-induced neuroinflammation and cognitive dysfunction.

Peripheral tissues release excessive pro-inflammatory mediators, reactive oxygen/nitrogen species, and cytotoxic proteases after burn injury. These excessive releases induce secondary neuroinflammation and immune imbalance, contributing to molecular and morphology changes and disruption of BBB structure (Shabir et al., 2018). The dysfunctions of BBB cause neuronal damage and brain edema (Barone et al., 2000). Magnetic resonance imaging (MRI) showed significant changes in brain within 3 days after burn injury, including brain swelling and lesions (Li et al., 2001), changes in cerebral blood flow (Li et al., 2009), and pathological manifestation of nerve necrosis and vacuolation (Li et al., 2001; Gatson et al., 2009). Third-degree skin burn injury in combination with smoke inhalation produce massive cerebral hemorrhaging, BBB dysfunction characterized by congested and dilated blood vessels, as well as neutrophil infiltration into the brain (Randolph et al., 2019). Dynamic contrast enhancement MRI (DCE-MRI) allows for the detection of BBB disruption in hippocampus as well as gray and white matters, demonstrating its association with cognitive decline (Verheggen et al., 2020). Behavioral studies have also showed long-term cognitive defects are associated with the destruction of BBB structure in post-burn animals (Swann et al., 2007; McColl et al., 2008; Terao et al., 2008).

The destruction of endothelial barrier is an important part in the progression of brain edema. The loss of Claudin5 (CLDN5) in tight junctions between BECs can lead to the compromise of BBB, resulting in cerebral edema and neural cell death (Yang et al., 2020). The increased permeability of BBB after severe burns in the rat model may also be related to the decreased expression of zonula occludens1 (ZO-1) in tight junctions (Liu et al., 2011). In rats with burn injury, the expression of hypoxia inducible factor-1 (HIF-1α) was upregulated, which consequently enhanced endothelial cell permeability through downregulation of Claudin5, ZO-1, VE-cadherin (Jiang et al., 2021). Umbilical cord-derived mesenchymal stem cells can protect the integrity of BBB after burns by decreasing the IL-6 and IL-1β level, protecting the tight junction, as well as reducing transcytosis and neuroinflammation (Yang et al., 2020). Matrix metalloproteinases (MMPs) also play a key role in the occurrence of BBB dysfunction after severe burn injury owing to the responses of BECs, microglia, and astrocytes to pathological conditions. MMPs were upregulated after burn injury, causing extracellular matrix (ECM) remodeling by breaking down the major basal lamina components, including fibronectin, laminin, and collagen IV (Hosomi et al., 2005). The levels of MMP-9 are particularly elevated after burn injury and cause subsequent cerebral edema (Hosomi et al., 2005; Berger et al., 2007; Swann et al., 2007). The neuro-mediator nitric oxide (NO) as a necessary medium for memory acquisition and consolidation is related to the long-term potentiation in hippocampus after severe burn injury (Walrath et al., 2022). Inhibition of NO can impair the acquisition of different learning tasks (spatial, association, avoidance) (Callow et al., 2021). One study found third-degree burn injury contributed to immediate reduction of NO in brain and the loss of working memory over the next few days. Long-term administration of NO synthase inhibitor L-NAME can block the expression of working memory during object recognition task (Bingor et al., 2020). Another study also reported that cognitive dysfunction was related to increased expression of tissue and urokinase plasminogen activators following peripheral thermal injury (Patel et al., 2008).

The transporters of BBB are functionally diverse and the transition of substances through BBB are bidirectional or unidirectional. The transporters of BBB may be reduced by burn injury, causing abnormal transportation of many molecules, such as choline, interleukin-1 family, glucose, triiodothyronine, TNF-α, enkephalins, and low-density lipoprotein receptor-related protein 1 (LRP-1) (Mooradian et al., 1991; Moinuddin et al., 2000; Banks et al., 2001; Wilson and Matschinsky, 2020). The mismatches between the brain requirement and BBB provision result in the dysfunctions of CNS. For example, the inhibition of LRP-1 contributes to the accumulation of Aβ peptides (Jaeger et al., 2009), which may result in memory impairment. The metabolism and transportation of glucose are closely related to cerebral blood flow (Wilson and Matschinsky, 2020), which may be interrupted by burn injury. Meanwhile, burn injury reduced the turnover of cerebrospinal fluid (CSF) (Damkier et al., 2013; Ma et al., 2017), which could be associated with memory defects. Perivascular transporting is more active in slow-wave sleep (Xie et al., 2013), but it may be disturbed with sleep disorder due to burn injury.

The dysfunctions of NVU are closely related to the decline of cognitive functions (Toth et al., 2017). The interactions among cells in NVU regulate the contraction and relaxation of blood vessels, the conduction of neurotransmitters, and maintain the stability of BBB functions and the internal environment (Chow et al., 2020). In the case of post-burn cerebral hypoperfusion, abnormal mechanisms of intercellular signal transduction and neurovascular coupling in NVU would develop. Although all the cell types of NVU communicate directly with BECs, the astrocytes and pericytes are the most important cells in inducing the formation of BBB from BECs and in regulating the structure and functions of BBB and NVU-BEC communication (Toth et al., 2017). After burn injury, the astrocytes may become hypertrophic and exhibit a more reactive morphological phenotype, with an increased expression of neuroinflammatory genes, increased oxidative metabolism, and altered regulation of glutamate (Walrath et al., 2022). The pericytes are critical to the integrity of BBB (Kurmann et al., 2021). The loss of pericytes leads to severe alterations in blood flow, which correlates with cognitive disruption (Montagne et al., 2020). To better understanding the role of pericytes in the CNS, a pericyte-specific *Cre* mouse model, was generated by utilizing a double-promoter approach with the platelet-derived growth factor receptor-β (Pdgfrb) and chondroitin sulfate proteoglycan-4 (Cspg4) promoters. Concomitant BBB dysfunction, severe loss of blood flow, rapid neuron loss, and neurodegeneration, as well as cognitive and behavioral changes, were found in pericyte-specific *Cre* mice (Nikolakopoulou et al., 2019). Pericytes are also critical for regulating transport systems (Kurmann et al., 2021); however, how these transport systems change after burn injury remains largely unknown.

In brief, the post-burn secondary inflammation in brain is closely related to the destruction of BBB structure, while the dysfunctions of BBB can aggravate the inflammatory responses. Their synergistic effects lead to cognitive dysfunctions after burn injury.

#### Hormonal imbalance

The HPA axis is a three-organ hormone cascade and feedback loop that is responsible for the level of circulating cortisol *in vivo*. Burn injury can induce the activation of neurons in the paraventricular nucleus of hypothalamus, which is the highest subcortical center of autonomic nerves, an important contact point of limbic system and reticular structures, and the excitation of pituitary endocrine system (Shafia et al., 2017). This activation can lead to the secretion of cortisol by adrenal glands (Shafia et al., 2017). Cortisol interacts with its peripheral receptors, including mineralocorticoid receptors and glucocorticoid receptors. These receptors regulate the hypothalamus-pituitary-adrenal (HPA) axis through a negative central feedback loop (Figure 4). HPA axis is the primary regulating mechanism for hormonal stress response, and its activity is associated with cognitive function (Miranda et al., 2019). Glucocorticoid receptors have been found in multiple regions of brain which are relevant to cognition, namely, hippocampus, amygdala, and prefrontal cortex (Shafia et al., 2017). Hippocampus is highly sensitive to stress and hypoxia, which makes it vulnerable to the burn event (Kim and Diamond, 2002). Stress can affect the proliferation, differentiation, maturation, survival and activation of newborn neurons (Schoenfeld and Gould, 2012), while the neurons in the dentate gyrus facilitates regulating the response of HPA axis to stress. This feedback loop is a sensitive mechanism because its alteration can produce dysregulation of HPA axis activities, causing several physical and psychological phenotypes, such as PTSD (Morris et al., 2012). A study of HPA axis in post-burn children reported that the adrenocorticotropic hormone-adrenal feedback loop was interrupted (Palmieri et al., 2006). Another meta-analysis also suggested that dysregulation of HPA axis is linked to poorer cognitive functions (Gardner et al., 2019).

Burn injury can cause hormonal changes in HPA axis, and changes in endogenous catecholamine levels (Caldwell et al., 1999). Temporary adrenocortical insufficiency has been observed in early stage of burn injury (Fuchs et al., 2007). After the initial burn injury, hyper catabolism occurs (Chance et al., 1989; Jeschke et al., 2008). This high metabolism is partly mediated by catecholamines and is related to the severity of burn injury (Wilmore et al., 1974). After severe burn injury, catecholamines in blood serum were sharply increased, leading to hyperdynamic circulation (Wilmore and Aulick, 1978). Patients with prolonged abnormal cortisol can lead to reduced hippocampal volume and defects in hippocampus-dependent memory tasks, as compared to controls with normal cortisol concentrations (Lupien et al., 2005).

To sum up, burn injury can lead to dysregulations of HPA axis and abnormal secretions of cortisol and other hormones. These dysregulations can cause a variety of psychological disorders, such as PTSD or MDD, as well as abnormalities in hippocampal morphology and functions, resulting in cognitive impairment such as memory defects.

## Clinical presentations and outcomes of post-burn cognitive sequelae

### Memory defects after burn injury

Memory is one of the most important components in the complex physiological process of cognition, and memory defects have an important impact on quality of life (QoL) of burn injured patients (Whyte et al., 2011). However, the clinical sequelae and potential comorbidity of memory defects following burn injury were seriously underestimated (Van Loey et al., 2003). A study found that cognitive impairments such as memory defect were neglected in post-burn rehabilitated patients without direct injury of brain (Woods et al., 2011), and study also observed significant abnormalities on electroencephalography in the post-burn patients (Wolters et al., 2013). The post-burn patients scored lower on the admission cognitive Functional Independence Measure (FIM) than did other rehabilitation groups (including groups with spinal cord injury, amputation, poly-trauma with multiple fracture, and hip replacement) (Purohit et al., 2014). The cognitive FIM includes five domains: memory, verbal comprehension, verbal expression, social interaction, and problem solving, each on an ordinal scale of 1–7, with a maximum total score of 35. In one study, memory scores showed the greatest difference between the post-burn and the other rehabilitation groups(5.1 ± 1.7 *vs.* 5.6 ± 1.5, *P* < 0.001) (Purohit et al., 2014). Burn injury can disrupt the cognitive process of autobiographical memory (Willebrand et al., 2002). A cue task found post-burn group recalled specific memories (an event that lasted less than a day) much more slowly compared with normal control group (Stokes et al., 2004). Memory defects seem to account for overall post-burn cognitive impairments (Clouston et al., 2022).

The elderly population is at higher risk of burn injury and its neurological sequelae due to more comorbidities. One previous study found that 11% of the burn injured patients were diagnosed with dementia at admission, but 18% of them were diagnosed with dementia at discharge (Holmes et al., 2017). In addition, patients with severe burn injury are more likely to be hospitalized in the intensive care unit (ICU), having a higher risk of long-term cognitive impairments with common symptoms of memory defects (Duggan et al., 2017). The memory defects became the most distressing problems in these patients discharged from ICU (Pandharipande et al., 2013; Duggan et al., 2017), which affected up to two-thirds of patients (Wolters et al., 2013) and last for at least 2 years (Watson et al., 2018).

### Psychological disorders after burn injury

Psychological function is also an important component of cognition. Symptoms such as PTSD, depression, anxiety, delirium, and hallucinations (Logsetty et al., 2016), may appear within hours to weeks after burn injury, affecting nearly 20% of post-burn patients (Patterson et al., 1993). An estimated 10–15% of these patients would continuously suffer from such symptoms, although most patients gradually recovered in one year after burn injury (Stoddard et al., 1989).

Post-traumatic stress disorder and depression are the two most common psychological disorders after burn injury (Palmu et al., 2011a), of which PTSD was particularly severe in hospitalized post-burn patients (Koenen et al., 2017). The prevalence of PTSD in post-burn patients is 7–45% (Dahl et al., 2016), being 35.1, 33.3, 28.6, and 25.4% at 1, 6, 12 and 24 months after injury, respectively (McKibben et al., 2008), and even lasting for the first 2 years (Dahl et al., 2016). The fully developed PTSD syndrome has a delayed onset, but individual symptoms of PTSD developed earlier in post-burn patients. The presenting symptoms include those that fulfill PTSD criteria C (i.e., emotional numbness and avoidance symptoms, such as an inability to recall events and estrangement from people) and PTSD criteria D (i.e., increased arousals such as heightened irritability and hypervigilance) (Giannoni-Pastor et al., 2016; Ali and Ali, 2022). A recent systematic review has shown predictive factors for PTSD in burn victims, such as the risk of life threat, severe pain, unmarried status, intrusive symptoms, substance abuse, depression, anxiety, previous mental diagnosis, and poor economic conditions (Giannoni-Pastor et al., 2016). Meta-analyses have shown that PTSD patients have hypocortisolism, in the form of abnormal cortisol levels (Schumacher et al., 2019; Pan et al., 2020). A common explanation for this phenomenon is increased negative feedback sensitivity (Fuchs et al., 2007; Daskalakis et al., 2013). This would imply dysregulation of the HPA axis and the hypocortisolemic pattern, which is consistent with the pathophysiological mechanisms of post-burn hormonal imbalance described previously. Hypocortisol may contribute to the chronic re-experiencing of traumatic situations. In particular, the administration of hydrocortisone can reduce PTSD symptoms and incidence (Kothgassner et al., 2021). Moreover, in patients with PTSD, cortisol was negatively associated with intrusive symptoms as well as avoidance, hyper-arousal, and numbness symptoms (Castro-Vale et al., 2016; Paggiaro et al., 2022). Therefore, glucocorticoid supplementation may have a therapeutic effect on PTSD after burn injury.

Depression always co-exists with PTSD (van Loey et al., 2012) after burn injury. One study showed that 52% post-burn patients with PTSD suffered from major depression disorder (MDD) (Rytwinski et al., 2013). Another study found about 25% of post-burn patients developed depressive symptoms three weeks later (Wiechman et al., 2016). In the study utilizing structural interviews, 4–10% of adult post-burn patients met the diagnosis criteria for severe depression within one year, and about 12.5% developed symptoms of depression 2 years later (Thombs et al., 2006). About 20–30% of post-burn patients sustained to suffer from depression for up to 20 years (Lawrence et al., 2016). The prevalence of depression in inpatients was even higher, ranging from 4 to 26% in self-reporting questionnaires (Thombs et al., 2006). In contrast to post-burn PTSD, what is involved in the development of depression is an increase in cortisol levels, which is also mainly due to the dysregulation of the HPA axis. The use of glucocorticoid (GR) antagonists in the treatment of depression has shown good therapeutic efficacy and safety (Mikulska et al., 2021; Flannery et al., 2022).

Anxiety is also prevalent among burn patients, with general anxiety being the most prevalent one due to the pain and stress caused by burns. Burn patients also suffer a type of anticipatory anxiety as a result of prolonged pain. Mismanagement of pain and anxiety in burn patients can result in fear, insomnia, depression, and inability to deal with the burn injury (Fardin et al., 2020). Delirium and hallucinations are common disorders in burn patients as well. Patients diagnosed with delirium have a higher mortality rate than non-delirium patients, requiring prolonged hospitalization as well as intensive care in the hospital (Mohseni Moallem Kolaei et al., 2021). Since changes in the HPA axis and hormones after burn injury are distinct in the types of psychological disorders, it is of great significance to explore the pathophysiological mechanisms deeply and combine them with large-scale clinical studies to clarify the development of psychological disorders impacted after burn injury.

## The management of post-burn cognitive sequelae

Cognitive impairments are closely related to the QoL of post-burn patients, so early identification and intervention of the impairments are essential. At present, most studies have been undertaken in experimental animals, or on the early clinical identification and interventions.

The limited studies on experimental animals mainly focus on the interventions of neuroinflammation and neural apoptosis as they are significantly related to cognitive functions. One animal study found that the gelsolin treatment can protect brain from burn injury in mice (Zhang et al., 2011). Exogenous gelsolin infusion can partially reduce the brain inflammation and apoptosis, and enhance the functions of peripheral T lymphocytes to improve the survival rate of severely burn injured mice (Zhang et al., 2011). Estrogen has also been shown to significantly reduce the levels of inflammatory cytokines and improve the prognosis of post-burn animal models. The estrogen can protect cells from increased programmed cell death (Gatson et al., 2009). These studies have suggested gelsolin and estrogen may have a protective effect on the cognition of severely burn injured patients, however, further clinical research is demanded.

As addressed before, burn injury can cause the damages of BBB structure characterized by increased permeability and brain edema. These damages are partly related to the increased expression of MMP-9 and the decreased basal layer proteins (Reyes et al., 2009). Doxycycline (a direct inhibitor of MMP-9) and TNF-α neutralizing antibody can alleviate these damages (Reyes et al., 2009). TNF-α neutralization antibody plays a role in protecting cognitive functions by inhibiting the activity of MMP-9, reversing the basal protein losses caused by burn injury, and improving the integrity of BBB structure (Reyes et al., 2009). Burn injury leads to an increased permeability of cerebral vessels. Hyperosmotic saline may be a beneficial addition to burn resuscitation protocols since it seemed to essentially eliminate the albumin leakage in the cerebral vessels (Barone et al., 1997). Burn injured mice exhibited cognitive defects associated with inflammation, apoptosis, oxidative stress in the prefrontal cortex and hippocampus. Matrine, a quinolizidine alkaloid isolated from *Sophora flavescens*, can alleviate cognitive defects after burn injury through biological activities, such as anti-nociception, and anti-inflammation (Khan et al., 2020).

Clinical studies have focused more on the early identification, diagnosis and interventions of cognitive dysfunctions following burn injury. Variables including %TBSA, history of ventilation, the burn injury of head and neck, history of tracheotomy, and number of operations were found to be important predictors for cognitive outcomes (Whyte et al., 2011). Post-burn patients receiving rehabilitation intervention can not only improve their physical and psychological function, but also cognitive function, independent ability, and have a shorter length of stay (Vogler et al., 2009). Burn survivors can experience a high QoL with complete cognitive and neurological functions. These studies have indicated early interventions for cognitive reconstruction can improve the process of cognitive and emotional adaptation after burn events, therefore patients at risk of developing cognition dysfunctions should be identified early.

Patient-reported outcome measures (PROMs) can identify patient needs and therapeutic progress. The CARe Burn Scale can be used to measure the QoL after burn injury so as to facilitate rehabilitation (Griffiths et al., 2021). The FIM instrument has been extensively studied and validated in the inpatient rehabilitation setting, especially the cognitive and motor domains, as an outcome predictor for discharge from rehabilitation in burn patients (Gerrard et al., 2013). Cognitive FIM score on admission can be used as a screening tool to identify these at-risk patients and make appropriate referrals (Purohit et al., 2014). Regular cognitive and psychological assessments are essential during hospitalization. Researches demonstrated the administration of a retrospective pre-burn EQ-5D plus Cognition measure can be used as a reference point to monitor individual Health-related Quality of Life (HRQL) recovery (Boersma-van Dam et al., 2021b), and timely interventions may benefit post-burn recovery (Fauerbach et al., 2020), for example cognitive behavior interventions can prevent persistent cognition impairments (Nolen-Hoeksema et al., 2008). Post-burn patients can learn to question their automatic negative thoughts and replace them with more positive ones by adjusting cognitive control (Demeyer et al., 2012). DCE-MRI could be applied as a potential imaging screening method for early detection and intervention of post-burn cognition defects (Verheggen et al., 2020).

Studies have shown that psychological disorders, such as depression and PTSD, are important predictors of poor long-term QoL in post-burn patients (Corry et al., 2010; van Loey et al., 2012). Psychological interventions seem to be promising in improving long-term HRQL of post-burn patients (van Loey et al., 2012). Previous studies showed psychological care is recommendable to post-burn patients due to the high prevalence of psychological disorders (Wisely and Tarrier, 2001), but only 6% of burn survivors consulted a psychiatrist or psychologist after discharge from acute care (Wisely and Tarrier, 2001). At 6 months after burn injury, 27% of the burn victims reported clear needs for psychological or psychiatric cares (Palmu et al., 2011b), however, only less than 50% of post-burn patients with clear needs received psychological or psychiatric care after discharge, and none of them received periodic psychotherapy (Palmu et al., 2011b). Clinicians are therefore advised to screen inpatients for acute PTSD so as to identify high-risk groups of chronic PTSD (van Loey et al., 2012), and are advised to carry out prospective psychological symptom screening, clinical evaluation and interventions (Gardner et al., 2012). The use of standardized questionnaires and the assessments of dissatisfaction with body image during early follow-up may help to identify psychological depressive symptoms (Dahl et al., 2016). Non-pharmacologic interventions, such as virtual reality, hypnosis, Yoga can reduce the psychological cognitive sequelae (Gasteratos et al., 2022). A systematic review addressing different interventions found that cognitive-behavioral therapy achieved the best outcome in alleviating PTSD in burn patients, while hypnosis and an informational education program did not produce sufficient effects (Paggiaro et al., 2022). A series of psychosocial interventions and outcome tools for pediatric burns have been shown to achieve statistically significant effects, most of which focus on techniques to provide distraction in the form of VR in the acute recovery phase. Burn camps, cognitive behavioral therapy, and parent counseling are promising, but larger, robust studies are needed (Hornsby et al., 2020).

In brief, post-burn cognitive impairments have not been widely recognized. Diagnosis and treatment of psychological disorders have been partly addressed in clinic, while the diagnosis and treatment of memory impairments are quite deficient.

## Conclusion

Burn injury has high rates of morbidity, disability, and mortality, causing neurological sequalae of cognitive dysfunctions with memory defects as the main manifestation. Here, we review the manifestation of post-burn cognitive impairments, the mechanisms of memory defects, and the intervention options.

The cognitive dysfunctions following burn injury are related to the primary factors and secondary factors. The primary factors are mainly the influences of burn event itself and the subsequent treatment process. The secondary factors refer to the secondary brain damage indirectly caused by burns, such as the inflammatory reactions in the brain, BBB dysfunctions, and hormone imbalance. As part of the NVU, BBB is a highly complex interface critical to the nutrition and homeostasis of CNS and will undergo a myriad of changes following burn injury. The destruction of BBB can lead to cell death, structural and functional abnormalities in the brain, resulting in cognition defects. Cognitive dysfunction after burn injury is an issue that is often overlooked during the clinical management of burn patients. In addition, it is closely related to the prognosis and subsequent quality of life of burn patients. Therefore, the recognition of post-burn cognitive impairment by clinical medical personnel should be improved. In particular, psychological disorders after burn injury have been investigated in clinics by limited researches, while memory dysfunctions still lack adequate attention. Moreover, the existing studies generally describe the manifestations of cognitive impairment after burn injury, while lack insight into the mechanisms underlying its development. The knowledge of memory impairment remains to be deepened in terms of its mechanism and treatments.

Maintaining the structure and functions of NVU and BBB, and early recognizing the disruptions of BBB can interfere with the occurrence and progression of cognitive dysfunctions following burn injury. Future studies should focus on exploring the effects of burn injury on NVU, especially on the structural and functional changes of BBB. Tools for early identification of cognitive dysfunction and the long-term intervention options should also be applied. In animal studies, more randomized controlled studies on the mechanisms of cognitive impairment and interventions after burn injury should be conducted; in clinical studies, large-scale sample-size research should be performed to gain insight into the composition, mechanisms and intervention of cognitive impairment. Mechanisms of cognitive dysfunction should be explored in terms of morphology molecular biology, neurobiology, and behavior so as to provide intervention targets and treatment options for cognitive dysfunctions after burn injury.

## Authors contributions

CX and JH collected, analyzed, and interpreted the materials and wrote the manuscript. JH and ZY conceived and designed the study and revised the manuscript. YC was responsible for the schematic diagram within this article and took part in a part of the material collection. All authors read and approved the final manuscript.
